# Novel Type IV Monitoring Device With Advanced Oximetry Indicators Offers Accurate Diagnosis of Obstructive Sleep Apnea in Adults

**DOI:** 10.1111/crj.70176

**Published:** 2026-02-17

**Authors:** Xu Wu, Hailiang Qin, Huai Huang, Jing Jiang, Xiaodan Wu, Zilong Liu, Min Li, Shanqun Li

**Affiliations:** ^1^ Department of Pulmonary and Critical Care Medicine, Zhongshan Hospital Fudan University Shanghai China; ^2^ School of Health Science and Engineering University of Shanghai for Science and Technology Shanghai China; ^3^ School of Mechatronic Engineering and Automation Shanghai University Shanghai China

**Keywords:** obstructive sleep apnea, oxygen desaturation index, type IV sleep device

## Abstract

**Background:**

Type IV sleep monitors offer a low‐burden option for obstructive sleep apnea (OSA) screening, yet their accuracy is often limited by motion artifacts and variability in signal‐processing methods. The PM50‐B is a wrist‐worn Type IV device that combines high‐sampling‐rate (200 Hz) photoplethysmography (PPG)‐based oximetry with wrist actigraphy to reduce motion artifacts and employs an adaptive SpO_2_ waveform‐based desaturation detection algorithm. This study aimed to validate the diagnostic performance of the PM50‐B against reference sleep studies.

**Methods:**

In this prospective observational study, adults with suspected OSA underwent simultaneous overnight recording with the PM50‐B and a reference test: in‐laboratory polysomnography (Type I), unattended polysomnography (Type II), or Type III home sleep apnea testing (HSAT). Oximetry and actigraphy signals were processed using a standardized workflow incorporating motion‐artifact attenuation, signal stabilization, and sleep–wake estimation. From the SpO_2_ signal, hypoxemia metrics were derived, including the oxygen desaturation index ODI2.5_5 (≥ 2.5% desaturation lasting ≥ 5 s/h of total sleep time), cumulative time with SpO_2_ < 90% and < 95% (CT90, CT95), and lowest SpO_2_. Agreement with the reference apnea–hypopnea index (AHI) was assessed using intraclass correlation coefficients, and diagnostic accuracy was evaluated at clinically relevant AHI thresholds.

**Results:**

A total of 475 participants were analysed (Type I, *n* = 37; Type II, *n* = 32; Type III, *n* = 406). ODI2.5_5 showed moderate‐to‐good agreement with AHI (ICC = 0.710) and good discrimination for moderate‐to‐severe OSA (AHI ≥ 15 events/h), with an under the curve (AUC) of 0.925 (sensitivity 81.20%, specificity 91.00%). Diagnostic performance was consistent across reference modalities (AUC range, 0.928–0.983).

**Conclusion:**

The PM50‐B provides clinically acceptable accuracy for OSA screening when combined with a standardized signal‐processing approach, particularly in comparison with Type III HSAT. ODI2.5_5 emerged as the strongest diagnostic metric, while CT90, CT95, and lowest SpO_2_ provided complementary characterization of nocturnal hypoxemia.

## Graphical abstract

As a Type IV sleep monitoring device, PM50‐B provides clinically acceptable accuracy for OSA screening when combined with a standardized signal‐processing approach. Compared with conventional ODI calculation methods, the proposed desaturation detection framework—incorporating adaptive baseline definition, persistence criteria at desaturation onset, and stable recovery requirements—provides improved temporal consistency and reduces ambiguity in event identification. This physiologically grounded approach contributes to more robust alignment between oximetry‐derived indices and AHI‐based disease classification.

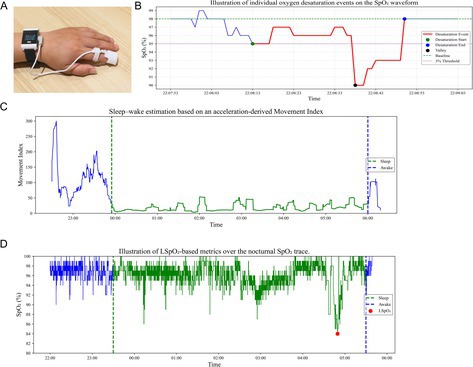

**Abbreviations**
AHI: apnea–hypopnea index
AUC: area under the receiver operating characteristic curve
CI: confidence interval
CT90: cumulative percentage of sleep time with SpO_2_ < 90%
HSAT: home sleep apnea testing
ICC: intraclass correlation coefficient
LoA: limits of agreement
LSpO_2_
: lowest peripheral oxygen saturation
MAE: mean absolute error
NPV: negative predictive value
ODI: oxygen desaturation index
OSA: obstructive sleep apnea
PPV: positive predictive value
PSG: polysomnography
RMSE: root mean square error
ROC: receiver operating characteristic
Se: sensitivity
Sp: specificity
SpO_2_
: peripheral oxygen saturation
TRT: total recording time
TST: total sleep time
*κ*
: Cohen's kappa coefficient

## 1 Introduction

Obstructive sleep apnea (OSA) is characterized by recurrent episodes of apnea and hypopnea accompanied by cyclic fluctuations in arterial oxygen saturation (SpO_2_) and is associated with substantial cardiovascular, metabolic, and neurocognitive morbidity [1]. Polysomnography (PSG) remains the reference standard for diagnosis; however, its accessibility is limited in many settings by high cost, resource intensity, and insufficient availability of sleep laboratories. To expand diagnostic capacity, home sleep apnea testing (HSAT) has increasingly incorporated Type III portable monitors (PMs), which typically record respiratory airflow, thoracoabdominal movement, and oxygen saturation and can be used to diagnose moderate‐to‐severe OSA in patients without significant comorbidities. Nevertheless, despite wider adoption, real‐world implementation still depends on appropriate equipment, trained support personnel, and standardized workflows, leaving a substantial proportion of OSA cases undiagnosed [2].

To further reduce testing burden and improve scalability, pulse oximetry is a widely available and cost‐effective technique routinely used in clinical practice [3]. Nigro et al. reported comparable areas under the curve (AUCs) and likelihood ratios when oximetry‐based screening was compared with a more comprehensive diagnostic approach [4]. Nevertheless, the reported performance of pulse oximetry for OSA detection varies substantially across studies, with large discrepancies in sensitivity and specificity when the apnea–hypopnea index (AHI) is used as the reference standard. Whether conventional oximetry parameters derived from Type IV devices can reliably predict AHI‐defined OSA remains controversial, and inconsistency persists regarding the optimal predictors and overall diagnostic accuracy of Type IV monitoring [5, 6]. Such variability is likely driven, at least in part, by motion artifacts that degrade signal quality and compromise desaturation detection, thereby hindering early identification and effective management of OSA [7].

The oxygen desaturation index (ODI) is a widely used oximetry‐derived marker of nocturnal hypoxemia and serves as a core index in out‐of‐center sleep assessment [8]. ODI‐based approaches, particularly when optimized for desaturation depth and duration, have shown promising performance for OSA screening in adults, including those with comorbidities [9]. However, prior reviews have reported wide ranges in specificity (47.5%–98%) and sensitivity (32%–98.5%), depending on pretest probability and the selected AHI threshold [10]. A key challenge is whether ODI‐based algorithms can maintain adequate diagnostic accuracy under real‐world conditions. Reducing this variability requires improved characterization of desaturation patterns (e.g., event frequency, duration, and depth) and identification of the most informative and clinically complementary oximetry‐derived indices. Accordingly, we sought to (i) validate PM50‐B–based Type IV screening against reference sleep studies and (ii) identify an optimal set of oximetry‐derived indicators for detecting suspected OSA in adults.

## 2 Materials and Methods

### 2.1 Study Design and Participants

This prospective, single‐center validation study evaluated the performance of the PM50‐B wrist‐worn oximetry system against reference sleep studies, including in‐laboratory polysomnography (Type I/II PSG) and Type III HSAT. The study was conducted at Zhongshan Hospital from April 2023 to May 2024 and was approved by the Institutional Ethics Committee (B‐2022‐264R) in accordance with the Declaration of Helsinki. Written informed consent was obtained from all participants.

**FIGURE 1:**
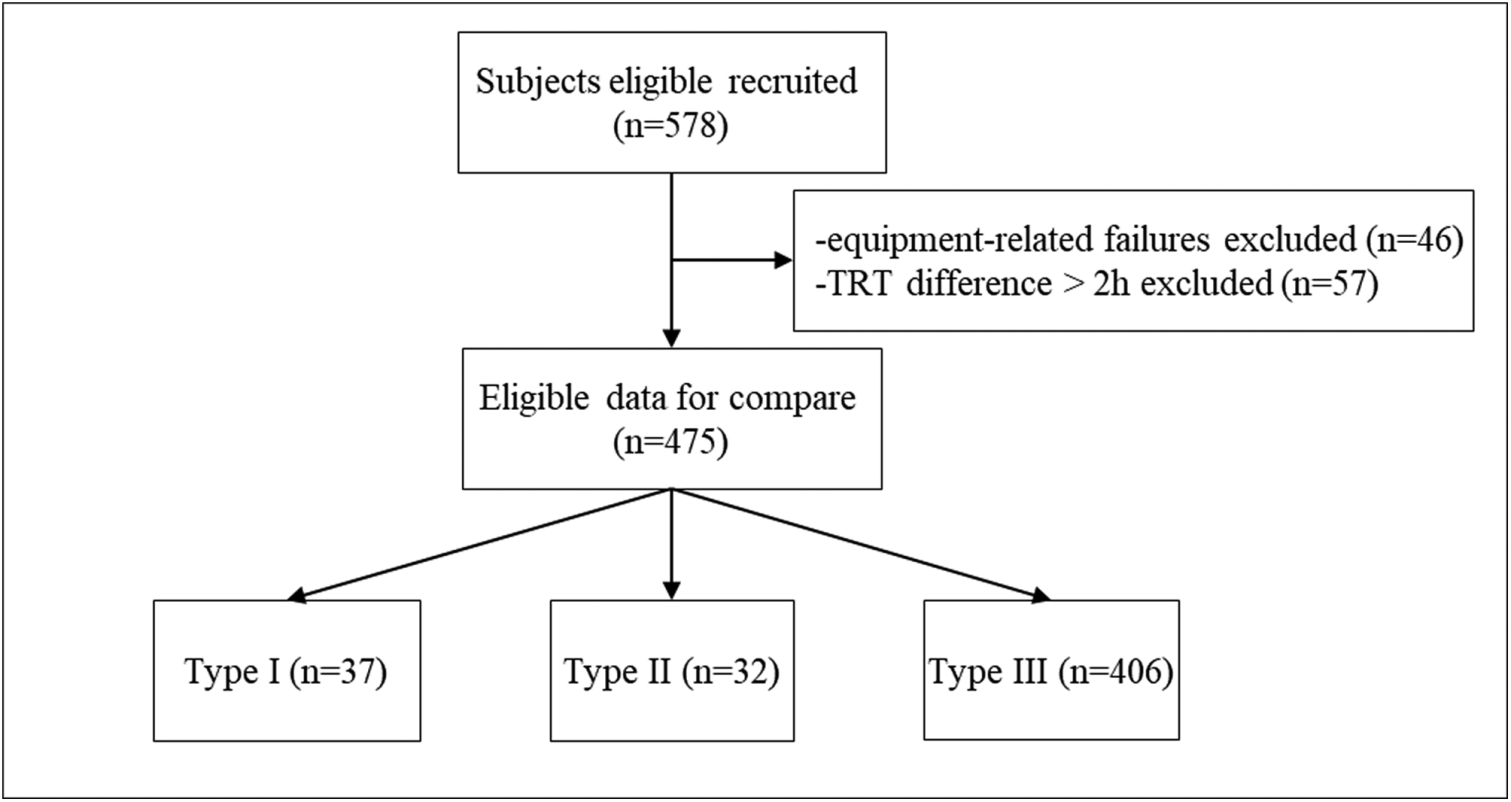
Flow diagram of participant recruitment. TRT, total recording time.

Participants were consecutively recruited from individuals referred to the sleep laboratory for overnight diagnostic evaluation because of symptoms suggestive of OSA, including habitual snoring, witnessed apneas, nocturnal choking, unrefreshing sleep, or excessive daytime sleepiness. No additional pre‐screening beyond routine clinical criteria was applied. Inclusion criteria were: (1) age ≥ 16 years; (2) ability to tolerate overnight monitoring; (3) intact and transparent fingernails suitable for pulse oximetry; and (4) no alcohol consumption on the study day. Exclusion criteria were based on known diagnoses or documented clinical history prior to the sleep study: (1) unstable mental status; (2) nocturnal oxygen therapy or noninvasive ventilation; (3) chronic heart failure, implanted pacemaker/defibrillator, asthma, chronic obstructive pulmonary disease (COPD), or obesity hypoventilation syndrome; (4) respiratory or cardiovascular conditions that independently cause oxygen desaturation (e.g., active pulmonary infection, pulmonary fibrosis, pulmonary heart disease); and (5) previously diagnosed sleep disorders other than OSA (e.g., central sleep apnea, sleep‐related hypoventilation). Participants with < 180 min of valid PM50‐B data or < 180 min of valid PSG/HSAT data were excluded. The recruitment flowchart is shown in Figure 1.

### 2.2 Reference Sleep Studies and Scoring

Type I PSG (Embla N7000; Embla Systems, USA) recorded standard diagnostic channels, including EEG (F_3_M_2_, F_4_M_1_, C_3_M_2_, C_4_M_1_, O_1_M_2_, and O_2_M_1_), bilateral EOG, submental EMG, ECG, bilateral anterior tibialis EMG, nasal thermistor airflow, nasal pressure, thoracoabdominal respiratory inductance plethysmography (RIP), snoring, and body position. Studies were considered technically adequate if they contained ≥ 6 h of sleep and ≥ 90% availability of essential channels (EEG, airflow, RIP, and oximetry). Type II PSG (Curative SM2000C; Curative Medical Technology Inc., China) included frontal, central, and occipital EEG (FpZ, F_4_, C_4_, O_2_, A_1_, and A_2_), two EOG channels, chin EMG, ECG, nasal airflow, pulse oximetry, thoracoabdominal movement sensors, leg EMG, position sensors, and audio recordings. Type III HSAT (Acumen 7; Curative Medical Technology Inc., China) recorded nasal airflow, thoracic and abdominal respiratory‐effort belts, finger pulse oximetry, snoring, and body position. Because Type III HSAT does not include EEG, total sleep time (TST) could not be directly measured and was estimated by manual editing of total recording time, excluding artifact segments and presumed wake periods identified from body position and respiratory‐pattern abnormalities.

Two independent certified sleep technicians, blinded to the PM50‐B results, manually scored all PSG/HSAT recordings according to standard clinical criteria. Obstructive apnea was defined as a ≥ 90% reduction in nasal airflow for ≥ 10 s with continued respiratory effort. Hypopnea was defined as a ≥ 30% reduction in nasal signal excursions lasting ≥ 10 s accompanied by either ≥ 3% oxygen desaturation or an arousal (arousals applicable only to Type I/II PSG). For Type III studies, TST estimation followed the procedure described above. All devices were time‐synchronized with the hospital's central server prior to acquisition to ensure clock alignment; this step was used solely for timestamp consistency and did not alter raw signals or derived summary indices.

### 2.3 PM50‐B Device and Accuracy Validation

**FIGURE 2:**
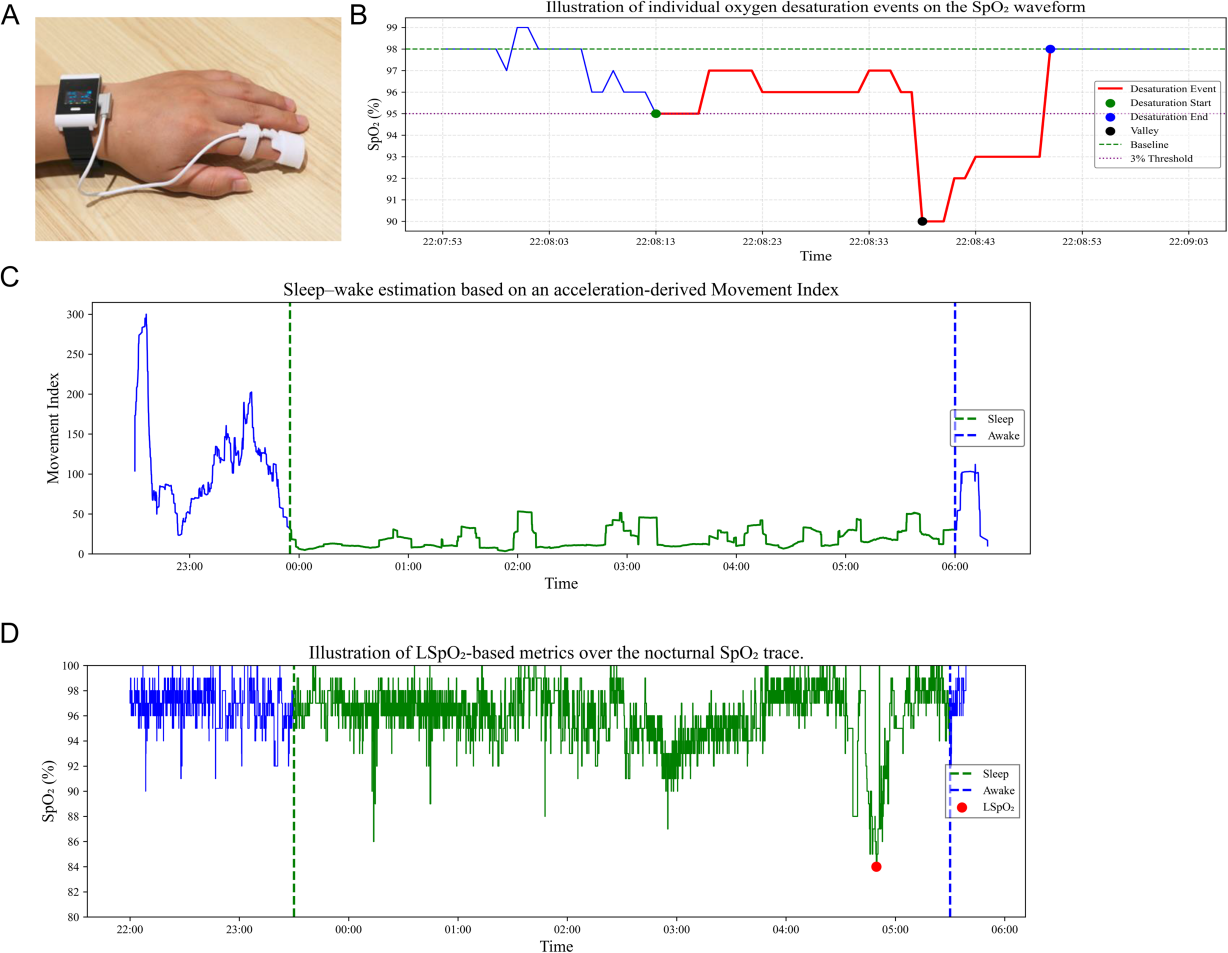
Overview of the PM50‐B device and signal‐processing illustrations. (A) PM50‐B worn on the wrist. (B) Illustration of individual oxygen desaturation events on the SpO_2_ waveform. (C) Sleep–wake estimation based on an acceleration‐derived Movement Index. (D) Illustration of LSpO_2_‐based metrics over the nocturnal SpO_2_ trace.

The PM50‐B (Berry Electronic Technology Co. Ltd., Shanghai, China) is a wrist‐worn pulse oximeter that integrates high‐sampling‐rate PPG and triaxial actigraphy. Raw PPG is sampled at 200 Hz and the device outputs computed SpO_2_ at 1 Hz. It includes a triaxial accelerometer for motion sensing and supports Bluetooth for wireless transfer; the device is lightweight and suitable for overnight monitoring (Figure 2A). All internal timestamps were referenced to the device clock synchronized to the hospital Network Time Protocol (NTP) server prior to recording.

SpO_2_ accuracy was validated in a controlled hypoxia study at Shenzhen University General Hospital (Nanshan, Shenzhen, China) following ISO 80601‐2‐61 requirements. Twenty‐four healthy adults underwent steady‐state hypoxia exposures spanning 70%–100% arterial oxygen saturation, with arterial blood gas co‐oximetry as the reference standard. Skin pigmentation was assessed using Fitzpatrick and Monk skin‐typing scales, including six participants with dark skin tones (Fitzpatrick V–VI/Monk 08–09). Under no‐motion conditions, PM50‐B achieved an ARMS of 2.23%, supporting reliability for nocturnal monitoring across skin tones.

### 2.4 Signal Processing and Derivation of Oximetry Metrics

All processing was performed offline in Python. PM50‐B provides three synchronized time series: (1) SpO_2_ at 1 Hz (derived from 200‐Hz PPG), (2) heartbeat event timestamps from PPG peak detection (timestamp resolution, 5 ms), and (3) a 1‐Hz movement signal computed as the vector magnitude of the triaxial accelerometer.

To mitigate motion artifacts, concurrent actigraphy was used to identify movement‐related disturbances. When a rapid SpO_2_ decline coincided with movement and was classified as artifactual, the apparent magnitude of the drop was algorithmically attenuated to reduce motion‐induced overestimation of desaturation depth and improve event specificity (Figure 2B). The 1‐Hz SpO_2_ series was smoothed using a 5 s moving average, and values were preserved at 0.1% resolution (rather than integer rounding) to form a high‐resolution pulse oximetry (HRPO) signal suitable for event detection.

Sleep–wake estimation was performed using the Movement Index algorithm, which computed the minimum sum of differences in acceleration along any two axes within a 10‐min sliding window. A sustained decrease in this index marked sleep onset, whereas a sharp increase indicated wake onset; a 5 s moving average was applied to reduce short‐term noise (Figure 2C). TST was defined as the interval between identified sleep onset and the final awakening. Only SpO_2_ samples within the validated sleep window were retained for downstream desaturation analysis to ensure that derived metrics reflected sleep‐related physiology.

LSpO_2_ was defined as the minimum SpO_2_ value recorded within the validated sleep window (TST). Prior to extraction, the SpO_2_ signal was subjected to motion‐artifact attenuation and smoothing to minimize the influence of transient signal dropouts or probe‐related artifacts. Only SpO_2_ samples within the verified sleep interval were considered, ensuring that LSpO_2_ reflects sleep‐related hypoxemia rather than recording artifacts. An example of LSpO_2_ is shown in Figure 2D.

Oxygen desaturation events were detected using an adaptive SpO_2_ waveform‐based desaturation detection algorithm that tracks rising and falling trends and identifies four ordered nodes on the SpO_2_ curve: P (Peak), the local maximum preceding a decline and serving as an adaptive baseline; S (Start), the first point at which a ≥ Δ% drop from P is observed and then confirmed by persisting for ≥ t_start seconds; V (Valley), the nadir of the desaturation; and E (End), the recovery point where SpO_2_ rises above a recovery threshold and remains elevated for ≥ t_recovery seconds. Events were retained only if the prespecified depth and duration criteria were satisfied within the validated sleep window.

Based on the results presented in the tables, the ODI was defined using a desaturation depth of ≥ 2.5% and a minimum duration of ≥ 5 s, expressed as the number of accepted desaturation events per hour of TST (ODI2.5_5). Complementary nocturnal hypoxemia metrics included minimum SpO_2_ during sleep (LSpO_2_) and cumulative time spent with SpO_2_ below fixed thresholds (CT90 and CT95, where applicable). All indices were calculated exclusively within the validated sleep window and were evaluated against the reference AHI.

### 2.5 Statistical Analysis

Descriptive statistics are presented as mean ± standard deviation or number (percentage), as appropriate. OSA severity was classified using standard AHI thresholds of ≥ 5, ≥ 15, and ≥ 30 events/h. The reference respiratory event index (AHI) was derived from the paired sleep study (PSG for Type I/II and Type III HSAT), using the modality‐specific scoring and TST estimation procedures described above.

Agreement between the primary oximetry‐derived indicator (ODI2.5_5) and the reference AHI was quantified using intraclass correlation coefficients (ICC; two‐way mixed‐effects model). Diagnostic classification performance at AHI thresholds of 5, 15, and 30 events/h was evaluated using sensitivity, specificity, accuracy, positive predictive value (PPV), negative predictive value (NPV), and Cohen's kappa. For ODI2.5_5, classification was performed using the corresponding fixed severity thresholds (i.e., ODI2.5_5 ≥ 5/15/30 events/h). For continuous hypoxemia indicators (CT90, CT95, and LSpO_2_, where applicable), discriminative performance was assessed at the same AHI thresholds using the same classification metrics, with optimal cutoffs determined as specified in the tables. All analyses were performed using Python 3.11 (SciPy and scikit‐learn) and SPSS Statistics 24 (IBM Corp., USA).

## 3 Results

### 3.1 Participant Flow and Baseline Characteristics

Of the 578 initially enrolled participants, 46 were excluded due to equipment‐related failures, including prolonged loss of the nasal airflow signal (> 1 h), insufficient total recording or sleep time (< 3 h), or persistently poor signal quality. An additional 57 participants were excluded because the total recording time differed by > 2 h between the PM50‐B and the paired reference study, precluding time‐aligned comparison. The final analytic cohort therefore comprised 475 participants, including Type I PSG (*n* = 37), Type II PSG (*n* = 32), and Type III HSAT (*n* = 406). Demographic and clinical characteristics are summarized in Table 1. Baseline nocturnal respiratory and oxygenation indices—including the AHI, oxygen desaturation indices, LSpO_2_, and cumulative hypoxemia metrics—are provided in Table S1.

**TABLE 1:** Baseline demographic and clinical characteristics of the study population.

Variables	All	Type I	Type II	Type III
N	475	37	32	406
Total recording time, h	9.13 ± 1.44	8.10 ± 1.30	8.64 ± 1.03	9.26 ± 1.44
Total sleep time, h	7.36 ± 1.37	7.23 ± 1.21	7.63 ± 1.20	7.35 ± 1.40
AHI, events/h	20.23 ± 21.49	26.16 ± 24.19	16.62 ± 20.15	19.97 ± 21.29
OSA severity (None/Mild/Moderate/Severe), %	32.8/23.6/17.1/26.5	29.8/10.8/18.9/40.5	37.5/28.1/18.8/15.6	32.8/24.4/16.7/26.1
Mean SpO_2_, %	96.48 ± 1.84	97.07 ± 1.60	96.55 ± 1.49	96.42 ± 1.88
LSpO_2_, %	76.02 ± 10.78	77.54 ± 11.42	79.34 ± 10.72	75.62 ± 10.70

*Note:* Values expressed as number (%) and mean ± standard deviation (SD). Mild OSA was defined as 5 ≤ AHI < 15 events/h; moderate OSA was defined as 15 ≤ AHI < 30; and severe OSA was defined as AHI ≥ 30 events/h.

Abbreviations: AHI, apnea–hypopnea index; LSpO_2_, the lowest peripheral oxygen saturation; ODI, oxygen desaturation index; OSA, obstructive sleep apnea.

### 3.2 Performance Screening of ODI Variants and Primary Metric Selection

We evaluated multiple prespecified parameterizations of the ODI derived from PM50‐B SpO_2_ (Table S2). Across the evaluated ODI definitions, Pearson correlations with the reference AHI ranged from *r* = 0.752 to 0.772, with corresponding *R* ^2^ = 0.566–0.597. Among ODI candidates, ODI2.5_5 demonstrated the most favorable overall profile and was therefore selected as the primary PM50‐B–derived metric for downstream analyses, showing *r* = 0.765, *R* ^2^ = 0.586, RMSE = 14.176 events/h, MAE = 8.983 events/h, and mean bias = −2.397 events/h. Agreement with AHI for ODI2.5_5 was moderate (ICC = 0.710), with Bland–Altman limits of agreement of 25.018 and −29.811 events/h (LoA range 54.830 events/h).

Compared with alternative ODI definitions (e.g., ODI2.5_6, ODI2.8_8, ODI2_6, ODI4_8, and ODI4_10), ODI2.5_5 provided the best combined performance in correlation, error, and agreement metrics (Table S2), supporting its use as the primary ODI configuration for AHI‐aligned Type IV screening in this cohort.

### 3.3 Agreement and Discrimination of PM50‐B–Derived ODI2.5_5

**FIGURE 3:**
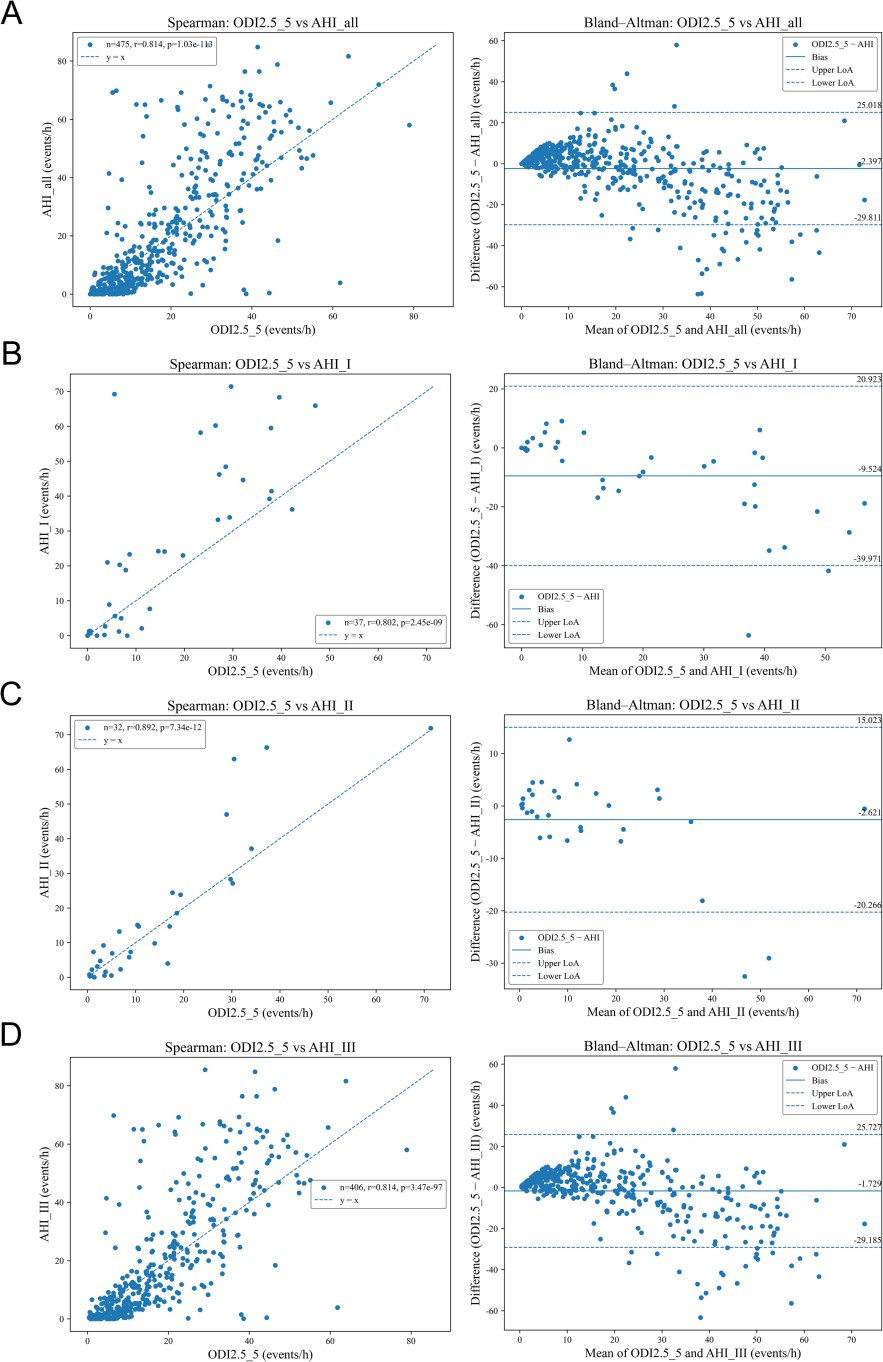
Spearman correlation scatter plots (*r*) and Bland–Altman plots comparing PM50‐B–derived ODI2.5_5 with the reference apnea–hypopnea index (AHI): (A) ODI2.5_5 vs. overall AHI, (B) ODI2.5_5 vs. Type I PSG‐AHI, (C) ODI2.5_5 vs. Type II PSG‐AHI, and (D) ODI2.5_5 vs. Type III HSAT‐AHI.

Bland–Altman analyses showed consistent agreement between PM50‐B–derived ODI2.5_5 and the reference AHI across modalities (Figure 3). The mean differences (ODI2.5_5—AHI) were −9.524 events/h for Type I PSG (95% limits of agreement, −39.971 to 20.923), −2.621 events/h for Type II PSG (−20.266 to 15.023), and −1.729 events/h for Type III HSAT (−29.185 to 25.727). In the overall cohort (*N* = 475), the mean bias was −2.397 events/h with 95% limits of agreement −29.811 to 25.018.

**FIGURE 4:**
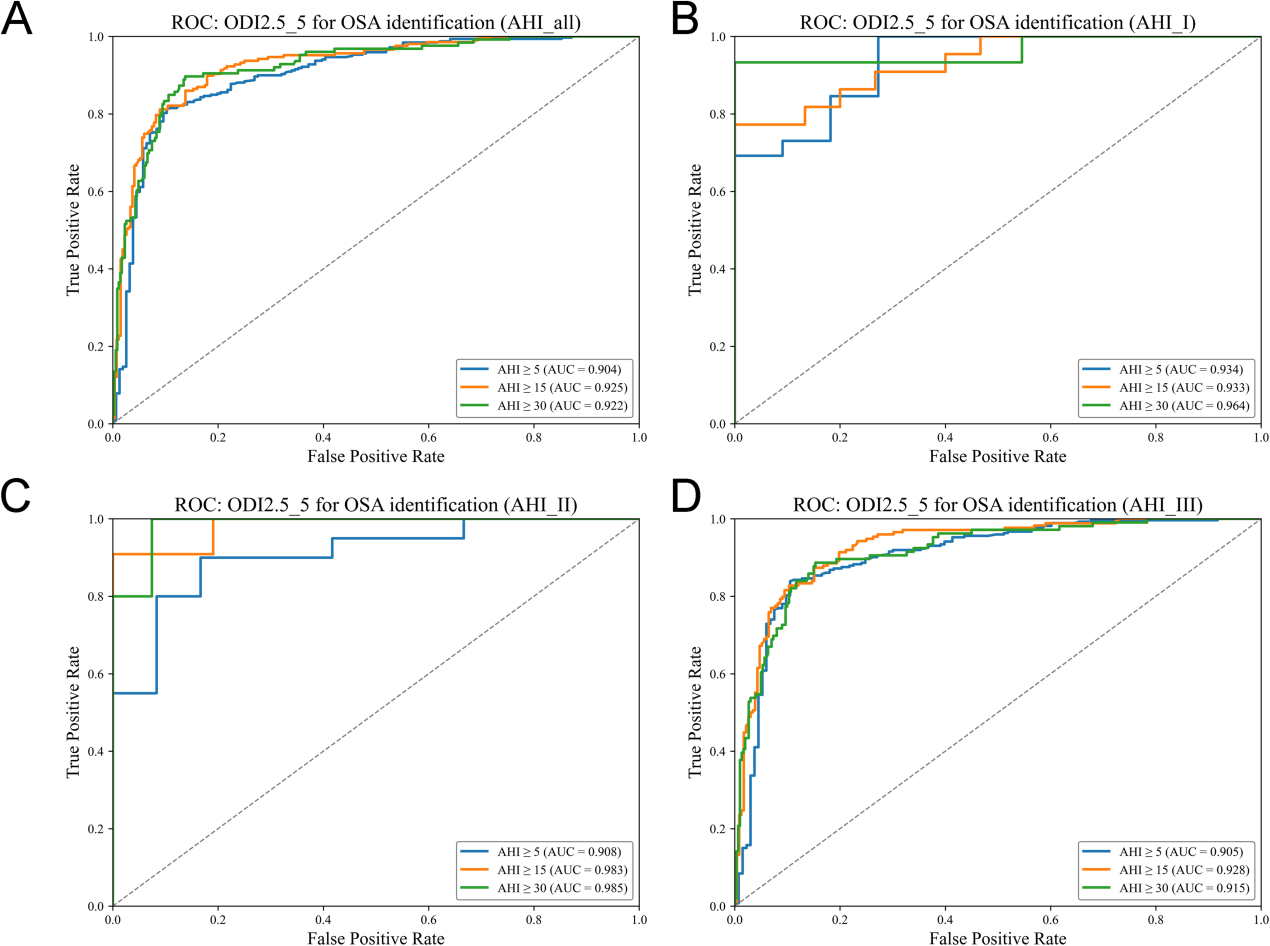
Receiver operating characteristic (ROC) curves showing the diagnostic accuracy of PM50‐B–derived ODI2.5_5 for identifying OSA at AHI thresholds of 5, 15, and 30 events/h, in (A) the overall cohort and (B–D) device‐stratified subgroups (Type I PSG, Type II PSG, and Type III HSAT).

Receiver operating characteristic (ROC) analyses demonstrated good discrimination across standard clinical severity thresholds (Figure 4). In the overall cohort, the AUCs were 0.904 for AHI ≥ 5, 0.925 for AHI ≥ 15, and 0.922 for AHI ≥ 30. Device‐stratified analyses showed AUCs of 0.934/0.933/0.964 (Type I), 0.908/0.983/0.985 (Type II), and 0.905/0.928/0.915 (Type III) for AHI ≥ 5/15/30, respectively.

### 3.4 Classification Performance and Complementary Hypoxemia Metrics

Using fixed cutoffs aligned with standard AHI thresholds, PM50‐B–derived ODI2.5_5 achieved strong classification performance for OSA across AHI thresholds, with full sensitivity, specificity, PPV, NPV, accuracy, and Cohen's kappa (*κ*) reported in Table 2. Specifically, for identifying moderate‐to‐severe OSA (AHI ≥ 15 events/h), ODI2.5_5 achieved sensitivity 86.96%, specificity 83.58%, accuracy 85.05%, and *κ* 0.70. For severe OSA (AHI ≥ 30 events/h), sensitivity was 62.70%, specificity 94.84%, accuracy 86.32%, and *κ* 0.62 (Table 3).

**TABLE 2:** Performance of oximetry indicators for diagnosing OSA.

Indicator	Severity	Cutoff	Sensitivity	Specificity	PPV	NPV	Accuracy	Cohen's kappa
ODI2.8_8	All	≥ 5.00	90.28%	69.23%	85.71%	77.70%	83.37%	0.61
	Moderate and severe	≥ 15.00	79.23%	91.79%	88.17%	85.12%	86.32%	0.72
	Severe	≥ 30.00	50.79%	97.71%	88.89%	84.62%	85.26%	0.56
ODI2.5_6	All	≥ 5.00	94.98%	55.77%	81.45%	84.47%	82.11%	0.56
	Moderate and severe	≥ 15.00	86.47%	85.45%	82.11%	89.11%	85.89%	0.71
	Severe	≥ 30.00	58.73%	95.42%	82.22%	86.49%	85.68%	0.60
ODI2.5_5	All	≥ 5.00	95.30%	53.21%	80.64%	84.69%	81.47%	0.54
	Moderate and severe	≥ 15.00	86.96%	83.58%	80.36%	89.24%	85.05%	0.70
	Severe	≥ 30.00	62.70%	94.84%	81.44%	87.57%	86.32%	0.62
ODI2_6	All	≥ 5.00	98.75%	38.46%	76.64%	93.75%	78.95%	0.44
	Moderate and severe	≥ 15.00	93.72%	73.13%	72.93%	93.78%	82.11%	0.65
	Severe	≥ 30.00	75.40%	91.69%	76.61%	91.17%	87.37%	0.67
ODI4_10	All	≥ 5.00	72.10%	94.23%	96.23%	62.29%	79.37%	0.59
	Moderate and severe	≥ 15.00	50.24%	97.39%	93.69%	71.70%	76.84%	0.50
	Severe	≥ 30.00	21.43%	99.14%	90.00%	77.75%	78.53%	0.27
ODI4_8	All	≥ 5.00	73.98%	94.23%	96.33%	63.91%	80.63%	0.61
	Moderate and severe	≥ 15.00	54.59%	97.01%	93.39%	73.45%	78.53%	0.54
	Severe	≥ 30.00	26.98%	98.85%	89.47%	78.95%	79.79%	0.33
LSpO_2_, %	All	≤ 81.00	81.82%	75.64%	87.29%	67.05%	79.79%	0.56
	Moderate and severe	≤ 78.00	86.96%	73.88%	72.00%	88.00%	79.58%	0.59
	Severe	≤ 75.00	91.27%	72.78%	54.76%	95.85%	77.68%	0.53
CT95, %	All	≥ 3.54	85.58%	78.21%	88.93%	72.62%	83.16%	0.63
	Moderate and severe	≥ 7.11	87.44%	76.49%	74.18%	88.74%	81.26%	0.63
	Severe	≥ 13.63	88.10%	78.22%	59.36%	94.79%	80.84%	0.57
CT90, %	All	≥ 0.68	77.12%	86.54%	92.13%	64.90%	80.21%	0.59
	Moderate and severe	≥ 1.87	76.81%	88.06%	83.25%	83.10%	83.16%	0.65
	Severe	≥ 2.72	89.68%	85.96%	69.75%	95.85%	86.95%	0.69

*Note:* Moderate OSA was defined as 15 ≤ AHI < 30; and severe OSA was defined as AHI ≥ 30 events/h.

Abbreviations: CTX, the cumulative percentage of the time spent at a SpO_2_ less than X%; LSpO_2_, the lowest peripheral oxygen saturation; NPV, negative predictive value; ODIX_Y, oxygen desaturation index with a ≥ X% drop sustained for Y seconds; OSA, obstructive sleep apnea; PPV, positive predictive value.

**TABLE 3:** ODI2.5_5 for Identifying OSA in three different sleep monitoring devices.

Type	Severity	Cutoff	Sensitivity	Specificity	PPV	NPV	Accuracy	Cohen's kappa
Type I	Mild	≥ 5	92.31%	72.73%	88.89%	80.00%	86.49%	0.668
	Moderate	≥ 15	72.73%	100.00%	100.00%	71.43%	83.78%	0.684
	Severe	≥ 30	46.67%	100.00%	100.00%	73.33%	78.38%	0.510
Type II	Mild	≥ 5	90.00%	83.33%	90.00%	83.33%	87.50%	0.733
	Moderate	≥ 15	90.91%	90.48%	83.33%	95.00%	90.62%	0.797
	Severe	≥ 30	80.00%	96.30%	80.00%	96.30%	93.75%	0.763
Type III	Mild	≥ 5	95.97%	48.87%	79.39%	85.53%	80.54%	0.504
	Moderate	≥ 15	88.51%	81.90%	78.57%	90.48%	84.73%	0.693
	Severe	≥ 30	64.15%	94.33%	80.00%	88.16%	86.45%	0.625

Abbreviations: NPV, negative predictive value; PPV, positive predictive value.

In addition to event‐count–based screening, continuous hypoxemia metrics provided complementary information. CT90 showed strong discrimination across severity thresholds, with an optimal cutoff of CT90 ≥ 2.72% for severe OSA (AHI ≥ 30). LSpO_2_ also demonstrated good diagnostic utility (Table 2), with optimal cutoffs of ≤ 81.00% (AHI ≥ 5), ≤ 78.00% (AHI ≥ 15), and ≤ 75.00% (AHI ≥ 30).

Device‐stratified classification results for ODI2.5_5 are summarized in Table 3. For Type III HSAT, ODI2.5_5 showed consistently high performance, achieving sensitivity/specificity of 95.97%/48.87% for AHI ≥ 5, 88.51%/81.90% for AHI ≥ 15, and 64.15%/94.33% for AHI ≥ 30, with corresponding accuracies of 80.54%, 84.73%, and 86.45% and *κ* values of 0.504, 0.693, and 0.625, respectively. For Type II PSG, accuracies were 87.50% (AHI ≥ 5), 90.62% (AHI ≥ 15), and 93.75% (AHI ≥ 30), with *κ* values of 0.733, 0.797, and 0.763, respectively. For Type I PSG, accuracies were 86.49% (AHI ≥ 5), 83.78% (AHI ≥ 15), and 78.38% (AHI ≥ 30), with *κ* values of 0.668, 0.684, and 0.510, respectively.

Collectively, these results indicate that PM50‐B–derived ODI2.5_5 provides robust screening performance across reference modalities, with particularly strong discrimination for moderate‐to‐severe OSA. Together with the complementary information from hypoxemia burden (CT90) and desaturation depth (LSpO_2_), the results highlight the value of the proposed PM50‐B–based analytical workflow for Type IV OSA screening.

## 4 Discussion

### 4.1 Main Findings

We developed an actigraphy‐informed workflow for the PM50‐B that integrates sleep‐window estimation (Movement Index), motion‐artifact mitigation using co‐located wrist actigraphy, and adaptive desaturation detection based on an adaptive SpO_2_ waveform‐based desaturation detection algorithm with systematic parameter screening. Using this approach, PM50‐B–derived ODI2.5_5 demonstrated moderate‐to‐good agreement with the reference AHI (ICC = 0.710) and consistent discrimination across standard clinical thresholds (AUC = 0.904 for AHI ≥ 5, 0.925 for AHI ≥ 15, and 0.922 for AHI ≥ 30), supporting ODI2.5_5 as a practical and physiologically plausible oximetry‐derived metric for adult OSA screening.

### 4.2 Comparison With Prior Work

The reported performance of ODI varies substantially across studies, largely because desaturation scoring rules differ in depth, duration, and baseline definition and are not standardized across devices. The proposed desaturation detection framework improves traceability by defining the baseline as the local peak (P) immediately preceding a decline and by enforcing a stable recovery end node (E), thereby reducing ambiguity in event boundaries that can inflate or suppress event counts.

Published ODI cutoffs are also influenced by cohort characteristics and target severity thresholds. For example, Ma et al. [11], in a pediatric population, reported that high specificity was primarily achieved for an ODI defined using a 4% desaturation threshold at an AHI cutoff of 15 events/h; however, pediatric findings may not directly generalize to adults. Tsai et al. [12] similarly demonstrated cohort‐specific ODI thresholds, with variable sensitivity and specificity depending on the selected desaturation definition and cohort characteristics, underscoring that no single ODI definition is universally optimal.

Benchmarking against open‐source implementations further contextualized our findings. Levy et al. [13] reported only moderate agreement between ODI and AHI (typically *R* ^2^ < 0.8), which is consistent with our observation that pobm‐based ODI benchmarks showed weaker agreement than the selected PM50‐B configuration (ODI2.5_5). These results indicate that refining desaturation depth–duration criteria and incorporating motion‐aware processing can yield measurable improvements in AHI‐aligned screening performance, even within the constraints of Type IV monitoring.

### 4.3 Clinical Implications

For Type IV PMs, optimal ODI definitions may depend on population characteristics and disease‐severity distribution [14], and accurate identification of mild OSA remains challenging. Within this context, ODI2.5_5 represents a compromise between sensitivity to short, shallow desaturations and robustness against motion‐related artifacts, yielding agreement levels that are clinically acceptable for screening rather than definitive diagnosis.

Beyond event‐based indices, selected continuous hypoxemia measures can provide complementary information when supported by the data. LSpO_2_ reflects the lowest observed oxygen saturation during sleep but is inherently sensitive to transient artifacts, as it depends on a single extreme value [15]. CT90, representing cumulative time spent with SpO_2_ < 90%, captures sustained hypoxemic burden and may be influenced by pulmonary reserve and ventilation–perfusion matching. Consistent with Julio et al. [16], CT90 may therefore offer additional clinical context, particularly in patients with more severe disease, when interpreted alongside ODI‐based screening results.

Commercial wearable devices increasingly incorporate nocturnal oximetry, heart rate, and movement signals and derive ODI using proprietary algorithms [17]. While wrist‐worn photoplethysmography is typically measured in reflectance mode, prior studies suggest that, under certain conditions, its performance can be comparable to transmissive PPG [18]. Higher PPG sampling rates may further improve temporal resolution for desaturation detection. Although heart‐rate variability (HRV) indices have been linked to cardiovascular outcomes in OSA [19] and explored for OSA risk prediction [20], we did not observe strong associations between HRV indices and AHI in our adult cohort, suggesting that the incremental value of HRV may depend on population risk profile and comorbidity burden rather than serving as a universal screening marker.

### 4.4 Limitations and Future Work

This study has several limitations. First, although participants were consecutively recruited from individuals referred for diagnostic sleep testing due to symptoms suggestive of OSA, subjective symptom questionnaires (e.g., the Epworth Sleepiness Scale) were not systematically collected; thus, our analyses emphasize objective nocturnal respiratory and oxygenation indices, and clinical interpretation for treatment decision‐making should be made with this in mind. Second, PM50‐B–derived metrics were evaluated at the study level rather than on an event‐by‐event basis, without direct temporal matching between individual desaturation events and scored apneas/hypopneas. Third, we did not differentiate obstructive from central respiratory events, and performance in populations enriched for central sleep apnea or complex sleep‐disordered breathing requires further validation. Finally, the Type I PSG subgroup was relatively small, and the study was conducted in a single‐center adult cohort, which may limit subgroup inference and generalizability to other settings (e.g., pediatrics or community screening). Future work should incorporate standardized symptom assessments, multicenter cohorts, and broader populations to further establish clinical utility.

## 5 Conclusion

In this prospective validation study, we demonstrated that a wrist‐worn Type IV oximetry system (PM50‐B), integrating high‐sampling‐rate PPG and wrist actigraphy, can provide a clinically meaningful assessment of OSA. A total of 475 adults were evaluated against reference sleep studies, including Type I/II polysomnography and Type III HSAT. Using an actigraphy‐informed processing workflow and an adaptive SpO_2_ waveform‐based desaturation detection algorithm, the ODI2.5_5 showed moderate‐to‐good agreement with the reference AHI and consistent diagnostic performance across standard clinical severity thresholds. These findings support ODI2.5_5 as a practical oximetry‐derived metric for adult OSA screening using a Type IV device.

Compared with conventional ODI calculation methods, the proposed desaturation detection framework—incorporating adaptive baseline definition, persistence criteria at desaturation onset, and stable recovery requirements—provides improved temporal consistency and reduces ambiguity in event identification. This physiologically grounded approach contributes to more robust alignment between oximetry‐derived indices and AHI‐based disease classification. In addition to event‐frequency metrics, cumulative hypoxemia measures such as CT90 and LSpO_2_ offered complementary information on nocturnal oxygenation status and disease severity, particularly in patients with more advanced OSA. Together, these indices illustrate the potential value of combining event‐based and burden‐based oximetry measures within a standardized analytical framework.

## Author Contributions

**Xu Wu:** writing – original draft, project administration, methodology. **Hailiang Qin:** methodology, investigation, formal analysis. **Huai Huang:** project administration, conceptualization. **Xiaodan Wu:** supervision, methodology, investigation, funding acquisition. **Jing Jiang:** writing – revision, formal analysis. **Shanqun Li:** review and editing, methodology, conceptualization, funding acquisition. **Zilong Liu:** investigation, formal analysis. **Min Li:** review and editing, methodology.

## Funding

This work was supported by the National Natural Science Foundation of China (82370088, 82470089, 82470090) and Data Sharing and Emulation of Clinical Trials (SHDC2025CCS044).

## Conflicts of Interest

The authors declare no conflicts of interest. Our automated parameter‐estimation algorithm is based solely on the SpO_2_ signal recorded by the PM50‐B device.

## Supporting information


**Table S1:** Distribution of AHI and oximetry‐derived indices.
**Table S2:** Comparison of PM50‐B–derived ODI definitions for AHI estimation

## Data Availability

Data with personal information removed can be obtained by corresponding authors.
