# Supplementary material for: Novel Type IV Monitoring Device With Advanced Oximetry Indicators Offers Accurate Diagnosis of Obstructive Sleep Apnea in Adults
Source: Clin Respir J. 2026 Feb 17;20(2):e70176. doi: 10.1111/crj.70176 (PMC12912879; doi:10.1111/crj.70176)
Supplement: Supplementary file 1 — Table S1: Distribution of AHI and oximetry‐derived indices. Table S2: Comparison of PM50‐B–derived ODI definitions for AHI estimation [file CRJ-20-e70176-s001.docx]

Table S1 Distribution of AHI and Oximetry-derived Indices

| **Variable** | **Median (Q1–Q3)** | **Mean ± SD** |
| --- | --- | --- |
| AHI (events/h) | 11.20 (2.55–33.90) | 20.23 ± 21.49 |
| Mean SpO_2_ (%) | 96.81 (95.72–97.74) | 96.48 ± 1.84 |
| LSpO_2_ (%) | 78.00 (65.00–85.00) | 76.02 ± 10.78 |
| ODI2_6(events/h) | 17.20 (8.66–30.21) | 20.23 ± 14.32 |
| ODI2.5_5 (events/h) | 13.95 (6.25–27.12) | 17.83 ± 14.38 |
| ODI2.5_6 (events/h) | 13.49 (6.03–26.12) | 17.33 ± 14.13 |
| ODI2.8_8 (events/h) | 10.44 (4.27–22.05) | 14.70 ± 13.23 |
| ODI4_10 (events/h) | 5.16 (1.54–14.45) | 9.74 ± 11.14 |
| ODI4_8 (events/h) | 5.50 (1.67–15.04) | 10.19 ± 11.50 |
| CT90 (%) | 1.02 (0.19–5.71) | 4.56 ± 7.49 |
| CT95 (%) | 8.03 (1.92–25.37) | 15.74 ± 18.26 |

LSpO_2_, the lowest peripheral oxygen saturation; ODIX_Y, oxygen desaturation index with a ≥X% drop sustained for Y seconds; CTX, the cumulative percentage of the time spent at a SpO_2_ less than X%.

Table S2 Comparison of PM50-B–Derived ODI Definitions for AHI Estimation

| **Metric** | **Sample Size** | **Pearson r** | **R²** | **RMSE** | **MAE** | **Bias** | **Upper LoA** | **Lower LoA** | **LoA Range** | **ICC** |
| --- | --- | --- | --- | --- | --- | --- | --- | --- | --- | --- |
| ODI2.5_5 | 475 | 0.765 | 0.586 | 14.176 | 8.983 | -2.397 | 25.018 | -29.811 | 54.830 | **0.710** |
| ODI2.5_6 | 475 | 0.765 | 0.586 | 14.309 | 8.984 | -2.894 | 24.601 | -30.388 | 54.989 | 0.705 |
| ODI2_6 | 475 | 0.752 | 0.566 | 14.267 | 9.621 | 0.003 | 27.996 | -27.991 | 55.987 | 0.697 |
| ODI2.8_8 | 475 | 0.772 | 0.597 | 15.093 | 9.141 | -5.528 | 22.027 | -33.084 | 55.112 | 0.692 |
| ODI4_8 | 475 | 0.766 | 0.587 | 17.766 | 11.336 | -10.039 | 18.720 | -38.799 | 57.519 | 0.640 |
| ODI4_10 | 475 | 0.764 | 0.584 | 18.150 | 11.656 | -10.489 | 18.574 | -39.552 | 58.127 | 0.627 |
| CT95 | 475 | 0.627 | 0.393 | 17.962 | 11.592 | -4.485 | 29.641 | -38.610 | 68.252 | 0.620 |
| CT90 | 475 | 0.700 | 0.490 | 23.183 | 16.179 | -15.667 | 17.860 | -49.194 | 67.053 | 0.436 |

ODIX_Y, oxygen desaturation index with a ≥X% drop sustained for Y seconds; CTX, the cumulative percentage of the time spent at a SpO_2_ less than X%.
